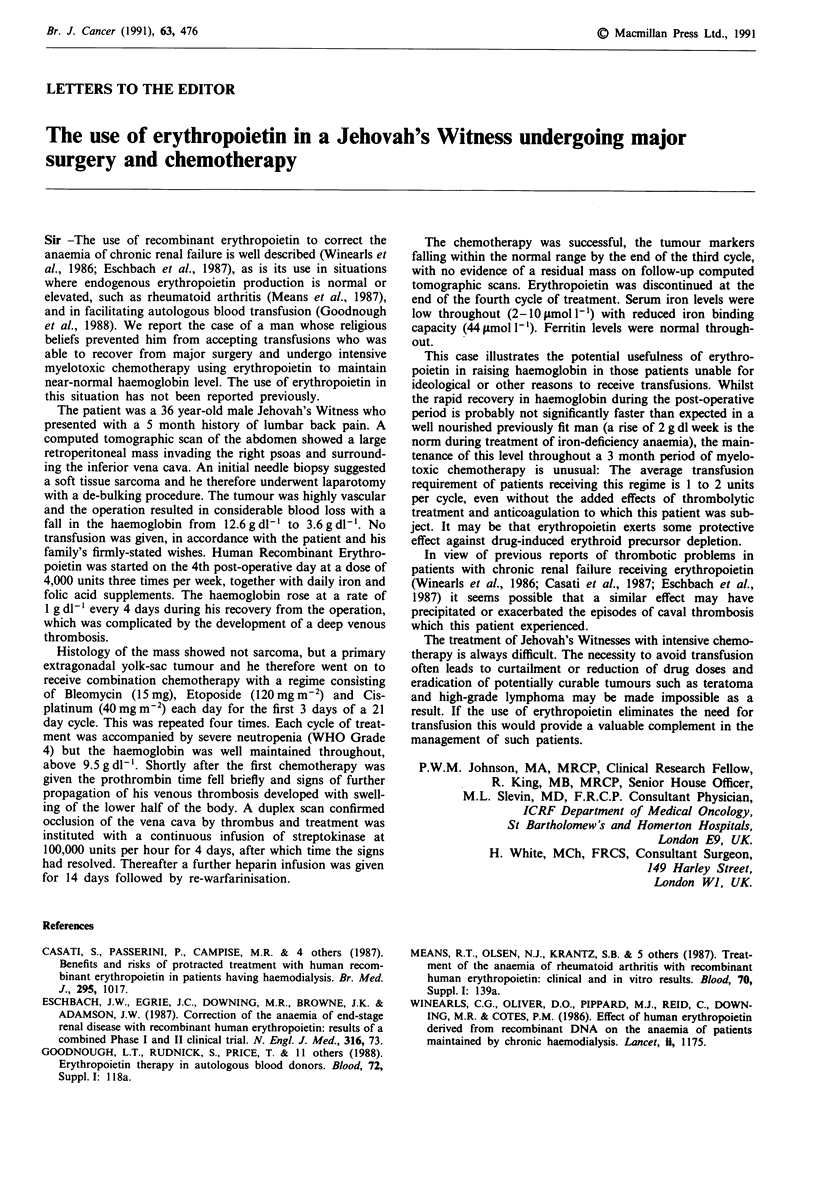# The use of erythropoietin in a Jehovah's Witness undergoing major surgery and chemotherapy.

**DOI:** 10.1038/bjc.1991.111

**Published:** 1991-03

**Authors:** P. W. Johnson, R. King, M. L. Slevin, H. White


© Macmillan Press Ltd., 1991

LETTERS TO THE EDITOR

### The use of erythropoietin in a Jehovah's Witness undergoing major surgery and chemotherapy

**Sir** –The use of recombinant erythropoietin to correct the anaemia of chronic renal failure is well described (Winearls *et al.*, 1986; Eschbach *et al.*, 1987), as is its use in situations where endogenous erythropoietin production is normal or elevated, such as rheumatoid arthritis (Means *et al.*, 1987), and in facilitating autologous blood transfusion (Goodnough *et al.*, 1988). We report the case of a man whose religious beliefs prevented him from accepting transfusions who was able to recover from major surgery and undergo intensive myelotoxic chemotherapy using erythropoietin to maintain near-normal haemoglobin level. The use of erythropoietin in this situation has not been reported previously.

The patient was a 36 year-old male Jehovah's Witness who presented with a 5 month history of lumbar back pain. A computed tomographic scan of the abdomen showed a large retroperitoneal mass invading the right psoas and surrounding the inferior vena cava. An initial needle biopsy suggested a soft tissue sarcoma and he therefore underwent laparotomy with a de-bulking procedure. The tumour was highly vascular and the operation resulted in considerable blood loss with a fall in the haemoglobin from 12.6 g $dl^{-1}$ to 3.6 g $dl^{-1}$. No transfusion was given, in accordance with the patient and his family's firmly-stated wishes. Human Recombinant Erythropoietin was started on the 4th post-operative day at a dose of 4,000 units three times per week, together with daily iron and folic acid supplements. The haemoglobin rose at a rate of 1 g $dl^{-1}$ every 4 days during his recovery from the operation, which was complicated by the development of a deep venous thrombosis.

Histology of the mass showed not sarcoma, but a primary extragonadal yolk-sac tumour and he therefore went on to receive combination chemotherapy with a regime consisting of Bleomycin (15 mg), Etoposide (120 mg $m^{-2}$) and Cisplatinum (40 mg $m^{-2}$) each day for the first 3 days of a 21 day cycle. This was repeated four times. Each cycle of treatment was accompanied by severe neutropenia (WHO Grade 4) but the haemoglobin was well maintained throughout, above 9.5 g $dl^{-1}$. Shortly after the first chemotherapy was given the prothrombin time fell briefly and signs of further propagation of his venous thrombosis developed with swelling of the lower half of the body. A duplex scan confirmed occlusion of the vena cava by thrombus and treatment was instituted with a continuous infusion of streptokinase at 100,000 units per hour for 4 days, after which time the signs had resolved. Thereafter a further heparin infusion was given for 14 days followed by re-warfarinisation.

The chemotherapy was successful, the tumour markers falling within the normal range by the end of the third cycle, with no evidence of a residual mass on follow-up computed tomographic scans. Erythropoietin was discontinued at the end of the fourth cycle of treatment. Serum iron levels were low throughout (2–10 $\mu$mol $l^{-1}$) with reduced iron binding capacity (44 $\mu$mol $l^{-1}$). Ferritin levels were normal throughout.

This case illustrates the potential usefulness of erythropoietin in raising haemoglobin in those patients unable for ideological or other reasons to receive transfusions. Whilst the rapid recovery in haemoglobin during the post-operative period is probably not significantly faster than expected in a well nourished previously fit man (a rise of 2 g dl week is the norm during treatment of iron-deficiency anaemia), the maintenance of this level throughout a 3 month period of myelotoxic chemotherapy is unusual: The average transfusion requirement of patients receiving this regime is 1 to 2 units per cycle, even without the added effects of thrombolytic treatment and anticoagulation to which this patient was subject. It may be that erythropoietin exerts some protective effect against drug-induced erythroid precursor depletion.

In view of previous reports of thrombotic problems in patients with chronic renal failure receiving erythropoietin (Winearls *et al.*, 1986; Casati *et al.*, 1987; Eschbach *et al.*, 1987) it seems possible that a similar effect may have precipitated or exacerbated the episodes of caval thrombosis which this patient experienced.

The treatment of Jehovah's Witnesses with intensive chemotherapy is always difficult. The necessity to avoid transfusion often leads to curtailment or reduction of drug doses and eradication of potentially curable tumours such as teratoma and high-grade lymphoma may be made impossible as a result. If the use of erythropoietin eliminates the need for transfusion this would provide a valuable complement in the management of such patients.

P.W.M. Johnson, MA, MRCP, Clinical Research Fellow,
R. King, MB, MRCP, Senior House Officer,
M.L. Slevin, MD, F.R.C.P. Consultant Physician,
*ICRF Department of Medical Oncology,*
*St Bartholomew's and Homerton Hospitals,*
*London E9, UK.*
H. White, MCh, FRCS, Consultant Surgeon,
*149 Harley Street,*
*London W1, UK.*

### References

CASATI, S., PASSERINI, P., CAMPISE, M.R. & 4 others (1987). Benefits and risks of protracted treatment with human recombinant erythropoietin in patients having haemodialysis. *Br. Med. J.*, **295**, 1017.

ESCHBACH, J.W., EGRIE, J.C., DOWNING, M.R., BROWNE, J.K. & ADAMSON, J.W. (1987). Correction of the anaemia of end-stage renal disease with recombinant human erythropoietin: results of a combined Phase I and II clinical trial. *N. Engl. J. Med.*, **316**, 73.

GOODNOUGH, L.T., RUDNICK, S., PRICE, T. & 11 others (1988). Erythropoietin therapy in autologous blood donors. *Blood*, **72**, Suppl. I: 118a.

MEANS, R.T., OLSEN, N.J., KRANTZ, S.B. & 5 others (1987). Treatment of the anaemia of rheumatoid arthritis with recombinant human erythropoietin: clinical and in vitro results. *Blood*, **70**, Suppl. I: 139a.

WINEARLS, C.G., OLIVER, D.O., PIPPARD, M.J., REID, C., DOWNING, M.R. & COTES, P.M. (1986). Effect of human erythropoietin derived from recombinant DNA on the anaemia of patients maintained by chronic haemodialysis. *Lancet*, **ii**, 1175.